# Cognitive Deficits in Myopathies

**DOI:** 10.3390/ijms21113795

**Published:** 2020-05-27

**Authors:** Eleni Peristeri, Athina-Maria Aloizou, Paraskevi Keramida, Zisis Tsouris, Vasileios Siokas, Alexios-Fotios A. Mentis, Efthimios Dardiotis

**Affiliations:** 1 Department of Neurology, Laboratory of Neurogenetics, Faculty of Medicine, University of Thessaly, University Hospital of Larissa, PC 41110 Larissa, Greece; eperiste@uth.gr (E.P.); aaloizou@med.uth.gr (A.-M.A.); viviker94@gmail.com (P.K.); tsouriszisis@me.com (Z.T.); bill_s1983@hotmail.com (V.S.); 2 Public Health Laboratories, Hellenic Pasteur Institute, PC 11521 Athens, Greece; mentisaf@gmail.com; 3 Department of Microbiology, Faculty of Medicine, University of Thessaly, University Hospital of Larissa, PC 41110 Larissa, Greece

**Keywords:** myopathies, dystrophies, cognitive deficits, behavioral indices, brain imaging indices

## Abstract

Myopathies represent a wide spectrum of heterogeneous diseases mainly characterized by the abnormal structure or functioning of skeletal muscle. The current paper provides a comprehensive overview of cognitive deficits observed in various myopathies by consulting the main libraries (Pubmed, Scopus and Google Scholar). This review focuses on the causal classification of myopathies and concomitant cognitive deficits. In most studies, cognitive deficits have been found after clinical observations while lesions were also present in brain imaging. Most studies refer to hereditary myopathies, mainly Duchenne muscular dystrophy (DMD), and myotonic dystrophies (MDs); therefore, most of the overview will focus on these subtypes of myopathies. Most recent bibliographical sources have been preferred.

## 1. Introduction

Myopathies represent a wide spectrum of heterogeneous diseases mainly characterized by the abnormal structure or functioning of skeletal muscle [1]; they can be hereditary or acquired, and can also manifest during the course of endocrine, autoimmune or metabolic disorders. Usually they worsen with time, and while their symptoms are not specific, muscle weakness, movement restriction (which can affect different muscle groups depending on the myopathy form, and which can also be transient) and also weakness and fatigue (among others) should raise the suspicion for a myopathy. The proximal muscles, namely, those of the shoulder, pelvis and upper thigh, are usually affected earlier in the disease course than the distal muscles, and give way to symptoms such as postural instability and inability to raise the hands or stand up from a sitting position. Myopathies eventually lead to muscular atrophy, with several patients being confined to wheelchairs, but they are typically not considered fatal; muscular dystrophy, a hereditary myopathy, is, however, considered to be severe [2].

Cognitive deficit is a general umbrella-term used to describe impairment(s) in an individual’s mental processes that underlie the acquisition of non-verbal and verbal information and knowledge, and drive how an individual interacts with the world. Cognitive function subdomains, including memory, attention, inhibition, problem solving and visual perception, have gained popularity in the field of neuropsychology. Importantly, though, cognition has been recently viewed through the lens of language processing, with fluency and narrative measures being widely-used in the field of neuropsychology. 

Due to the pathogenesis of myopathies being diverse and several systems being affected, cognitive deficits may also appear as a result of these diseases. Given the gap in the literature regarding cognitive defects within the context of myopathies, we have attempted to gather and present the studies on this matter and provide much-needed insight for clinicians handling these patients, who are often in need of multidisciplinary care.

## 2. Myopathy Categories and Associated Cognitive Deficits

There are several forms of myopathies, from genetic/hereditary ones to endocrine-related, to name a few. The aim of this review is not to provide an overview of myopathies, but rather to present the available studies on the relationships between some forms of myopathies and cognitive functions. Therefore, we have categorized this section based on a basic causal classification of myopathy types, and only present those for which studies exploring cognitive functions have been published, hoping not to overflow the reader with information not pertaining to the review’s purpose.

### 2.1. Genetic Myopathies 

Muscle cells need thousands of proteins in order to remain functional, so a great number of genes are implicated in their protein production. Hereditary or genetic myopathies arise as a result of a genetic defects leading to either the absence or the alteration of respective proteins vital for muscular function, and they differ in their clinical presentation due to their different genetic backgrounds. Many genetic myopathies have been well-described, but studies regarding concomitant cognitive deficits are very scarce; in fact, we only found relevant studies that focused on two types of genetic myopathies, besides the most well-known Duchenne muscular dystrophy and myotonic dystrophy.

#### 2.1.1. Duchenne Muscular Dystrophy

Muscular dystrophies are usually described separately from other myopathies, although they can be included in this umbrella-term due to the muscle weakness and wasting that they entail. In general, their main difference from what is typically perceived as a myopathy is that, while myopathies are caused by genetic defects in the contractile apparatus of muscles, muscular dystrophies are diseases of the muscle membrane and structural elements [3]. They are hereditary diseases, with the causative genes more or less identified. Their age of onset is usually in childhood and adolescence, although some forms affect younger or older individuals [2]. 

Duchenne muscular dystrophy (DMD) follows the X-linked recessive inheritance pattern. The affected gene in the X chromosome encodes the protein dystrophin, which is crucial for the musculature structure, but is also present in other tissues and the central nervous system (CNS) as well, mainly in the hippocampus, the cerebellum and the neocortex [4]. Clinically, it is characterized by severe, progressive and irreversible loss of muscular function, presented as predominantly proximal muscle weakness, eventual loss of ambulation, elevated serum creatine kinase (CK) levels, calf pseudohypertrophy and involvement of the cardiorespiratory system, leading to motor delays or regression [1,5]. Due to its cognitive aspects having been extensively studied, we deemed it important to include this in our review, although the taxonomy of myopathies/muscular dystrophy is generally not very clear in the particular disease.

The neurodevelopmental sequelae of DMD have long been known, and their prevalence is currently thought to be higher than once perceived [6], although global cognitive deficits are not noted in every patient. To further elucidate this phenomenon, Wingeier et al. (2011) studied a cohort of 25 boys with genetically confirmed DMD. The subjects underwent a detailed neuropsychological assessment and scored very low in a wide array of tests, including arithmetic and verbal fluency. The full scale intelligence quotient (IQ) was found one standard deviation (SD) lower than average, while verbal IQ received a stronger blow than non-verbal IQ [5]. This has been reciprocated in other studies that showed a delay in language milestone achievement in the mother tongue and poor narrative skills. Several other cognitive aspects also seem to be impaired in DMD, with studies showing deficits in working memory, attention and executive function [7,8,9,10,11,12,13,14,15]. For example, Kreis et al. (2011) reported deficits in verbal short-term memory and fluency, and visuospatial long-term memory, accompanied by a drop in full-scale IQ [4]. The hypothesis that these impairments may arise due to cerebellar dysfunction has also been stated, since this clinical image in DMD is similar to that of patients with lesions in the cerebellum [14,15,16].

However, as already mentioned, the non-progressive cognitive deficits that have been tied to DMD [17] do not lead to a distinct and homogenous phenotype across all DMD patients. No clear reason for this phenomenon has been pinpointed so far, but the pathogenesis seems to involve different dystrophin isoforms. For example, it has been shown that patients lacking the Dp140 isoform present important cognitive deficits [5]. Additionally, DMD has been linked to several neurobehavioral disorders also attributed to dysfunctional dystrophin isoforms. Banihani et al. (2015) conducted a retrospective cohort study with 59 boys with DMD. A full-scale IQ of <70 was reported in 27% of the patients, learning disability in 44% and intellectual disability in 19%; meanwhile, 32% carried a concomitant ADHD (attention-deficit/hyperactivity disorder) diagnosis, 15% had disorders of the autism spectrum and 27% presented anxiety. Of the children with learning disorders, 60% carried mutations affecting the Dp260 isoform or the 5′UTR region of Dp140, with the respective percentage for autism spectrum disorders being 77%, 50% for intellectual disability and 94% for anxiety. Furthermore, patients with mutations affecting the middle and 3′ end of the gene were also reported to present higher rates of cognitive impairment and ADHD, while the researchers also associated severe intellectual disability and ADHD with mutations in exon 19, a finding that, per the authors, has been replicated by other researchers [18]. In a different study, ADHD in DMD patients was also linked to Dp140 mutations, and mutations predicted to influence all dystrophin subtypes [19]. Interestingly, in a study of cerebellar and neocortical metabolite disorders in DMD patients, the Dp140 deficit was not associated with brain metabolism, thereby suggesting that the noted cognitive deficits of the lacking Dp140 were not mediated through the studied metabolites [4]. All in all, these results highlight the roles the specific isoforms affected in DMD play in cognitive functioning, further hinting that detecting the mutated isoform could predict cognitive impairment and that early intervention concerning mental functions is highly needed. 

The proposed mechanisms underlying the reported cognitive deficits within the context of DMD are versatile. Some attribute them to the different affected dystrophin isoforms and others to the involvement of dystrophin in embryonic development; others still imply an interplay between genes and non-genetic factors; one thing is for certain, no single mechanism has been so far identified. The involvement of the cerebellum has been postulated in some studies, given the similarities between the noted impairments in DMD—in particular, those pertaining to speech and verbal functions, with cerebellar lesions. To further examine this theory, Kreis et al. (2011) performed a metabolic analysis in the cerebellum and the temporo-parietal region of 12 and 8 DMD patients respectively, compared to 15 controls. They showed consistent choline deficits in both areas under study, and significant disorders concerning glutamate and N-acetyl compounds in the temporo-parietal regions. In their DMD cohort, total N-acetyl compounds in the temporo-parietal region were linked to verbal IQ and verbal short-term memory. On the other hand, choline and the putative general metabolic disorder were not found to be significantly associated with cognitive deficits, although the researchers mentioned that their reported choline deficit came in contrast to earlier similar studies that found an overabundance of choline in the cerebellum of DMD subjects [4].

As the aforementioned studies also suggest, boys with DMD are more susceptible to learning disability, and their verbal IQ seems to be more affected than nonverbal IQ. It has been reported that up to 40% of DMD patients had difficulty in reading and further demonstrated deficits in phonological awareness/processing, and in short-term verbal memory [18,19]. These academic difficulties seem to derive from learning disabilities such as dyslexia, and other cognitive deficits, such as working memory impairments [12]. Patients with DMD have been shown for instance to have learning difficulties qualitatively akin to subjects with developmental dyslexia (specific reading and writing difficulties, reduced automatized naming speed), although slightly less severe, and have also been shown to have difficulty with phonological processing similarly to a subgroup of individuals with dyslexia [20]. 

Concerning ADHD, it seems to be the neurobehavioral disorder most commonly associated with DMD [18,19]. More specifically, Banihani et al. (2015) reported ADHD in 32% of their 59 DMD patients [18], while Pane et al. (2012) reported the exact same percentage for their 103 DMD patients, which is almost four-fold the percentage for average school-aged children (8–10%). They also reported that deficits in attention, either combined with hyperactivity or not, were more frequent than hyperactivity alone [19]. Battini et al. (2018) have also recently studied 40 DMD boys without intellectual disability, and reported that several cognitive functions were affected, especially those that tapped on to multi-tasking, problem solving, inhibition and working memory, all of them being crucial subcomponents underlying goal-oriented behavior [21]. These deficits could explain the higher prevalence of ADHD in DMD, and to some extent, the higher rates of learning disorders as well.

Moving to autism spectrum disorders (ASD), it has been reported that their incidence in DMD ranges from 4% to 37% [22,23,24,25]. For instance, Banihani et al. (2015) reported a concomitant ASD diagnosis in 15% of their DMD patients [18], and Wu et al. (2005) had earlier expressed the notion that the co-occurrence of DMD and ASD in not coincidental [23]. These percentages are much higher than the latest estimates from CDC, where only 1 in 68 general-population children is thought to suffer from ASD [26,27]. In a similar vein, although most DMD boys seem to cope fairly well with their medical condition, emotional problems, such as anxiety, are also twice as likely to manifest in DMD patients [18].

#### 2.1.2. Myotonic Dystrophies

Myotonic dystrophies (DMs) follow the autosomal dominant mode of inheritance and affect several organs. Clinically, two subtypes are recognized: DM type 1 (Steinert’s disease), caused by a trinucleotide repeat expansion in the DMPK gene, and DM type 2, caused by a tetranucleotide repeat expansion in the ZNF9/CNBP gene [28]. Concerning their manifestation, both subtypes include myotonia (i.e., the prolonged muscle contraction that cannot be relaxed upon movement cessation), hence their name; muscular dystrophy; arrhythmias/cardiac conduction disorders; and cataracts. Additionally, involvement of the endocrine, gastrointestinal, respiratory and central nervous systems may also be present [29]. However similar in terms of symptoms, the clinical phenotypes of the two subtypes are distinct; in particular, DM1 is characterized mainly by facial and distal-predominant limb weakness, grip myotonia and no fluctuation, whereas DM2 by progressive proximal and distal limb weakness, and variable mild grip myotonia [28,30,31]. Finally, congenital myotonic dystrophy (CDM) usually manifests in the first month of life [32] and is considered to be the most severe form of DM1 [33]. 

As already mentioned, myotonic dystrophies 1 and 2 present distinct phenotypic differences, and these differences extend to the patients’ neuropsychological profiles as well. For this reason, we will explore the cognitive deficits noted in each of these subtypes separately.

Some studies on children with DM1 have associated the degree of cognitive impairment (lower IQ scores) with the number of the trinucleotide repeats, tied to maternal inheritance, also affecting the age of disease onset, although no such association was revealed for the neuromuscular involvement and the overall disease severity [34,35,36]. In a recently published retrospective study, 74 DM1 patients, 52 of them being affected by CDM, were evaluated. Seventy-four percent of the cases had maternal inheritance, with the number of trinucleotide repeats spanning from 143 to 2300. More than half of the patients presented some degree of cognitive delay, with a higher percentage noted in those with the congenital form, while the vast majority of the patients had some sort of cognitive, developmental or behavioral disorder. The researchers also mentioned that speech/language delay was often observed. Formal IQ testing was only available for a subgroup of the patients, and showed that most scored below average. Finally, ADHD and mood disorders also presented higher-than-average rates in the cohort, but patients with infantile DM1 had particularly high rates [32]. Woo et al. (2019) have also recently compared adult-onset and juvenile-onset DM1 in a cohort of 19 DM1 patients that underwent numerous neuropsychological tests. Verbal intelligence and verbal memory were significantly impaired in the juvenile group, while both groups performed equally well in performance intelligence and executive function tasks [37]. Deficits in verbal functions, which were more prominent in the juvenile cohort, might be indicative of a neurodevelopmental disorder in the earlier-onset subtypes.

Congenital DM1 is considered to be the most severe early form of DM1 and is often accompanied by cerebral atrophy and ventricular enlargement since birth. Besides the developmental milestones presenting considerable delay, all patients suffer from mental retardation with global learning difficulties [28]. In childhood-onset DM1, sometimes children first present cognitive symptoms, expressed as learning difficulties hinting towards mental retardation, before showing signs of muscular involvement [30]. In these patients, IQ scores were comparable to those of the general population and their learning problems seemed to stem from executive function deficits, alongside impairments in other cognitive domains, such as visual perception, memory (specifically in visuospatial recall and verbal memory) and constructional ability. Additionally, they tended to present signs of psychopathological disorders, such as ADHD and anxiety [34,38,39,40,41]. As expected, without a family history of MD, a diagnosis is difficult to be made, and the symptomatology of the children is usually only tied to MD after one of the parents is diagnosed with adult-onset DM1 [30]. Here, we would like to mention the entity of late-onset oligosymptomatic DM1, which is also characterized by mild symptomatology in earlier generations, with a worsened disease course in the generations to follow (especially, the third generation) [30]. These findings suggest that the age of onset of the disease is gradually set at an earlier time point as generations progress, and could be the outcome of more trinucleotide repetitions being gradually added; for example, congenital DM1 has been associated with extremely large repeat numbers [42].

In adult-onset DM1, structural and functional brain abnormalities have been noted. The most typical neuropsychological symptom seems to be reduced perception skills -an avoidance, therefore, of the disease’s other signs and symptoms. This can be accompanied by obsessive, compulsive, schizotypal, passive-aggressive and other emotional disorder characteristics [34]. Depressive symptoms pertaining to both childhood and adulthood DM1, are mostly the sequelae of the disease diagnosis and its psychological impact, as it often leads to a life of low quality [43], while daytime sleepiness is usually the consequence of physical disability and at times obstructive apnea. Structure-wise, brain MRI scans of DM1 patients showed diffuse white-matter alterations which were more prominent than atrophy [30,44]. 

The involvement of the CNS constitutes one of the main differences between DM1 and DM2. Individuals with DM2 may also present cognitive deficits, but these are milder than in DM1, and are considered as unusual occurrences [5]. Meola et al. (2003) conducted a positron emission tomography (PET) study on 21 DM1 and 19 DM2 patients that underwent cognitive assessment. They have noted cognitive deficits pertaining to frontal lobe dysfunction (planning and conceptual reasoning), with one test having significantly worse scores only for the DM1 cohort. They also reported reduced cerebral blood flow in the frontal, parietal and temporal lobes, which was linked to cognitive impairment [45]. The same group, in an earlier study with 20 DM1 and 20 DM2 patients, reported that two thirds of DM2 patients, compared to half of DM1 patients, presented visuospatial recall impairments, while a smaller percentage of DM2 patients had deficits in visuospatial construction. MRI scans were either normal or with non-specific white matter lesions in both cohorts, but the PET scans revealed a more diffuse hypoperfusion of frontal regions in DM1 patients [46], a finding that is in accordance with the general acknowledgement that cognitive deficits best characterize DM1 rather than DM2.

#### 2.1.3. Other Genetic Myopathies

Inclusion body myopathy with early-onset Paget disease and fronto-temporal dementia (FTD) is a rare hereditary disease caused by mutations mostly in the valosin containing protein (VCP) gene, with an autosomal dominant pattern of inheritance. As the name suggests, it is characterized by myopathy, firstly occurring in the proximal muscles and progressing to the extremities and other muscles. Half of the patients develop Paget disease of the bones, with skeletal pains, and one third develop the signs and symptoms of FTD, such as dysnomia, personality changes and attention deficits [47]. It is important to note that it has been reported for patients with no family history but with novel VCP mutations [48], so this should be taken into consideration in the differential diagnosis of patients with myopathy that present signs of FTD or Paget bone disease.

Muscle tissues require big amounts of energy to function, and so mitochondrial function is of paramount importance. Mitochondrial myopathies arise due to deficient oxidative phosphorylation in the mitochondria, which leads to a deficit in ATP and impaired skeletal muscle function. They are the results of mutations in genes implicated in mitochondrial function, and due to the solely maternal inheritance of mitochondrial DNA, this pattern of inheritance should be taken into consideration when investigating such cases. The symptoms vary depending on the different diseases and tend to affect multiple systems besides muscles [49]. One particularly interesting condition in terms of its cognitive phenotype is the mitochondrial encephalopathy, lactic acidosis and stroke-like episodes (MELAS) syndrome. MELAS usually occurs before the age of 40, but overall presents heterogeneity at its early stages. It is caused by specific mitochondrial DNA mutations and muscle biopsies show ragged red fibers [50]. Kraya et al. (2019) studied 10 patients with MELAS syndrome and found that they had lower scores than controls in the entirety of the neuropsychological tests. Specifically, significant differences were reported for tests assessing visual construction ability, visual and divided attention, and verbal fluency [51]. The cardinal symptoms of the syndrome, mainly the stroke-like episodes and the encephalopathy, can also manifest in altered mental status, and lead to gradual accumulation of a plethora of deficits in neurological functions. Eventually, 40% to 90% of the patients develop dementia, mainly as a result of the cortical lesions caused by the stroke-like episodes. Executive functions are also impaired, although neuroimaging studies have shown that the frontal lobe is not particularly affected, and that a generalized, possibly neurodegenerative process underlies cognitive dysfunction. Finally, the syndrome has also been reported to be accompanied by psychiatric disorders, such as depression, psychosis, anxiety and bipolar disorder [52].

A concise presentation of the main cognitive deficits noted in genetic myopathies can be found in Table 1.

### 2.2. Acquired Myopathies

#### Endocrine-Related Myopathies

It is widely known that endocrine malfunctions heavily impact muscle functions, with several endocrinopathies producing muscular symptoms; hypercortisolism and thyroid disorders are the entities most commonly associated with myopathy.

Steroid myopathy is the result of hypercortisolism, either via long-term exogenous corticosteroid administration or in Cushing’s syndrome, with glucocorticoid-induced myopathy being the commonest drug-induced myopathy [53]. Muscle weakness and atrophy mainly affect the proximal muscles and are accompanied by other typical hypercortisolism symptoms, such as body trunk obesity, virilization and cushingoid (“moon”) facies [54].

Hyperthyroid myopathy accompanies an over-function of the thyroid gland and leads to generalized muscle weakness with quickly setting fatigue, occasional mild muscle atrophy in proximal muscle zones and involvement of the ocular muscles [55]. Hypothyroid myopathy manifests in the setting of hypothyroidism and entails muscle rigidity, cramps and general weakness, pronounced in the extremities [2,56]. Both have also been tied to rhabdomyolysis, especially hypothyroidism, with elevated serum CK enzymes [55], while a similar entity has been described in aggressively-treated hyperthyroidism [57].

Due to the multifaceted nature of these diseases, it is hard to support the notion that a reported cognitive deficit is linked to the myopathy, and not to the overall endocrine disorder itself. These disorders almost always affect the CNS to a larger or smaller extent, with some conditions having neurological symptoms as cardinal symptoms.

In greater detail, a 2015 study on a pediatric population with thyroid disorders showed that impaired overall cognitive function may be tied to hypothyroidism myopathy, with deficits in attention, memory, arithmetic and verbal skills being mainly reported [57]. Mental retardation has also long been associated with severe hypothyroidism and is one of the main reasons as to why neonatal screening is being considered as a standard clinical practice, while the differential diagnosis of acquired cognitive deficits in older individuals typically includes thyroid function tests [58]. Similarly, individuals with hyperthyroidism and its associated myopathy may also present with altered mental status, emotional instability and confusion [57]. The specific symptoms are classically attributed to increased thyroid function.

Finally, hypercortisolism has also long been associated with a variety of neuropsychological disorders; emotional instability, cognitive impairment, depression and anxiety symptoms are frequently encountered. An excess in cortisol has been shown to induce structural changes in CNS regions such as the hippocampi, which can explain the cognitive symptoms associated with Cushing syndrome [59]. Since the muscular symptoms are only one aspect of the wide symptomatology of hypercortisolism, an attempt to examine the effect of this myopathy on cognition has not yet been made.

## 3. Conclusions

Cognitive deficits in myopathies do not seem to be that rare of an occurrence. Some muscular disorders present impairment in well-described cognitive functions, and others are also frequently tied to neuropsychological disorders, such as ADHD and anxiety. In certain diseases, especially the genetic ones, phenotypical variability is often the derivative of different isoforms being involved, or of the existence of certain proteins both in muscles and the CNS. In the majority of other myopathies, such as those tied to endocrine disorders, it is difficult to pinpoint the effect or the association between myopathy and cognitive impairment, given than most of the times, this impairment is considered a steady part of the symptom constellation of the disorder and is the direct result of the endocrine dysregulation. Regardless, studies on the matter are still lacking. This is understandable to a degree, given that the diseases in hand are not frequent and recruiting cohorts of patients big enough to provide the extraction of powerful results is hard. Additionally, the wide array of neurobehavioral assessment tools does not always help in classifying the results, as studies may use diverse IQ tests and neuropsychological batteries to test cognitive impairment. However, given the impact that cognitive impairment has on the life quality of a person already burdened by the muscular involvement itself, it is crucial that more research be conducted in the future, so that timely intervention may possibly preserve the individuals’ cognitive functions.

## Figures and Tables

**Table 1:** Cognitive deficits reported in genetic myopathies.

Genetic Myopathy	Associated Cognitive Deficits
Duchenne Muscular Dystrophy	Full-scale and verbal IQ decrease Language milestone achievement delay Working, verbal short-term and visuospatial long-term memory impairment Phonological awareness/processing deficits Learning difficulties (e.g., akin to dyslexia) High rates of ADHD and ASD
Myotonic Dystrophy 1	Full-scale IQ decrease Language milestone achievement delay Verbal memory deficits High rates of ADHD and mood disorders
Congenital Myotonic Dystrophy	Developmental milestone achievement delay Mental retardation Global learning difficulties
Myotonic Dystrophy 2	Planning and conceptual reasoning deficits Visuospatial recall impairment Milder cognitive deficits than in Type 1
MELAS Syndrome	Global cognitive deficits Visual construction, visual and divided attention, verbal fluency impairments High rates of dementia, anxiety, depression, psychosis and bipolar disorder

IQ: intelligence quotient; ADHD: attention deficit hyperactivity disorder; ASD: autism spectrum disorder; MELAS: mitochondrial encephalopathy, lactic acidosis, stroke-like episodes.
